# Factors contributing to deep supercooling capability and cold survival in dwarf bamboo (*Sasa senanensis*) leaf blades

**DOI:** 10.3389/fpls.2014.00791

**Published:** 2015-01-13

**Authors:** Masaya Ishikawa, Asuka Oda, Reiko Fukami, Akira Kuriyama

**Affiliations:** ^1^Functional Plant Research Unit, Division of Plant Sciences, National Institute of Agrobiological SciencesTsukuba, Japan; ^2^Graduate School of Advanced Science and Technology, Tokyo Denki UniversityHiki-gun, Japan

**Keywords:** bamboo, cold hardiness, deep supercooling, freezing injury, ice barrier, *Sasa senanensis*, sclerenchyma, tissue unit

## Abstract

Wintering *Sasa senanensis*, dwarf bamboo, is known to employ deep supercooling as the mechanism of cold hardiness in most of its tissues from leaves to rhizomes. The breakdown of supercooling in leaf blades has been shown to proceed in a random and scattered manner with a small piece of tissue surrounded by longitudinal and transverse veins serving as the unit of freezing. The unique cold hardiness mechanism of this plant was further characterized using current year leaf blades. Cold hardiness levels (LT_20_: the lethal temperature at which 20% of the leaf blades are injured) seasonally increased from August (−11°C) to December (−20°C). This coincided with the increases in supercooling capability of the leaf blades as expressed by the initiation temperature of low temperature exotherms (LTE) detected in differential thermal analyses (DTA). When leaf blades were stored at −5°C for 1–14 days, there was no nucleation of the supercooled tissue units either in summer or winter. However, only summer leaf blades suffered significant injury after prolonged supercooling of the tissue units. This may be a novel type of low temperature-induced injury in supercooled state at subfreezing temperatures. When winter leaf blades were maintained at the threshold temperature (−20°C), a longer storage period (1–7 days) increased lethal freezing of the supercooled tissue units. Within a wintering shoot, the second or third leaf blade from the top was most cold hardy and leaf blades at lower positions tended to suffer more injury due to lethal freezing of the supercooled units. LTE were shifted to higher temperatures (2–5°C) after a lethal freeze-thaw cycle. The results demonstrate that the tissue unit compartmentalized with longitudinal and transverse veins serves as the unit of supercooling and temperature- and time-dependent freezing of the units is lethal both in laboratory freeze tests and in the field. To establish such supercooling in the unit, structural ice barriers such as development of sclerenchyma and biochemical mechanisms to increase the stability of supercooling are considered important. These mechanisms are discussed in regard to ecological and physiological significance in winter survival.

## Introduction

Dwarf woody bamboos are the dominant component of forests and grasslands in Japan (Numata, 1979). *Sasa senanensis* (Fr. and Sav.) Rehd. is one such evergreen woody species belonging to Bambusodae supertribe, Poaceae. It occurs mainly on the Japan sea side of Japan from southern Honshu to Hokkaido and reaches a mean height of 1.5–2.0 m in the forest understory (Ohshima, 1962; Lei and Koike, 1998). Its distribution range is known to match the areas having a mean snow deposit depth exceeding 50 cm (Suzuki, 1961). *S. senanensis* is probably one of the most cold hardy species (Ishikawa, unpublished) among the woody bamboo species that distribute worldwide from tropical to cool temperate regions or high elevations (Soderstrom, 1981). The cold hardiness level in mid-winter expressed as the lowest temperature that can be tolerated without any injury is about −15°C and ranks the third after *Sasa kurilensis* and *Sasamorpha borealis* among the major dwarf bamboo species that occur in the cool temperate region in Japan (Ishikawa, unpublished; Konno and Sakai, 1975).

*S. senanensis* is known to be unique with respect to its freezing behavior (freezing strategy): most of its live tissues such as leaf blades, leaf sheaths, culms and rhizomes employ deep supercooling as the mechanism of cold hardiness (Ishikawa, 1984). Most typically, the live cells (e.g., mesophyll cells) in the leaf blades of *S. senanensis* remain unfrozen by stable deep supercooling until −20°C. This is in contrast to the majority of the wintering leaves of cold hardy evergreen woody plants and herbaceous plants, which undergo extracellular freezing, except for a cold hardy palm, *Trachycarpus fortunei* which also employs deep supercooling as the cold hardiness mechanism (Larcher et al., 1991; Ishikawa et al., 2009). Cryomicroscopic observation of the freezing process in *S. senanensis* leaf blades revealed that a small piece (rectangular shaped) of tissue surrounded by longitudinal and transverse veins functions as the unit of deep supercooling (Ishikawa, 1984). And yet, factors influencing the deep supercooling capability in the leaf blades of *S. senanensis* remain to be elucidated, such as how the supercooling capability changes seasonally and depending upon the leaf position in a shoot, how stable the supercooling is upon prolonged exposure to subfreezing temperatures and whether the tissue intactness is required for the supercooling capability. These questions have been frequently addressed with the deep supercooling capability in the xylem ray parenchyma cells.

The mechanism of deep supercooling has been mainly studied using xylem ray parenchyma cells of cold-hardy woody stems (George and Burke, 1977; Hong and Sucoff, 1982; Wisniewski, 1995). A problem with xylem is that only ray parenchyma remain supercooled and the majority of neighboring tissues such as xylem vessels, cambium and phloem tissues are frozen. This makes it difficult to conduct pin-point biochemical and molecular analyses of the tissues that deep-supercool (Fujikawa et al., 2009). Direct observation of deep supercooling/freezing in the xylem ray parenchyma is practically impossible without sectioning the tissues (Neuner et al., 2010; Pramsohler et al., 2012) and may induce artifacts. In *S. senanensis*, most of the live tissues such as culms, leaf sheaths and rhizomes also remain deep-supercooled (Ishikawa, 1984). In this sense, *S. senanensis* is a deep-supercooling plant. Among these tissues, the leaf blade may serve as the best material as the majority of its live cells including mesophyll cells remain deeply supercooled and is thin enough to be translucent and allow direct cryomicroscopic observation. These properties potentially allow various analyses and experimentation to be easily conducted with only deep-supercooling tissues or without damaging the tissues, making this material a good system for the study of deep supercooling. Thus, factors and mechanisms involved in the deep supercooling capability of leaf blades should be studied in detail.

When considering the wintering capacity of plants in the field, cold hardiness determined in laboratory freeze tests using a single condition may not be sufficient. Prolonged exposure to low temperature is often important for winter survival determination (Gusta et al., 2009). Electrolyte leakage tests employed in cold hardiness determination of *Arabidopsis* often provide only averaged information whilst visual injury determination provides more localized or tissue specific injuries in plants. Thus, we determined cold hardiness or freezing injury by visual observation of the plants and checked the effects of prolonged exposure to subfreezing temperatures besides the normal laboratory freeze tests. These may help resolve the seeming discrepancy in the levels of cold hardiness in leaf blades (−20°C vs. −15°C) between our previous study (Ishikawa, 1984) and that of Konno and Sakai (1975).

The objective of this study is to further characterize the nature of deep supercooling in leaf blade tissues of *S. senanensis* with the following design. First, involvement of tissue units in the maintenance and breakdown of deep supercooling in the leaf blade is carefully studied. Secondly, the stability of supercooling and importance of ice barriers are addressed. Thirdly, the effects of leaf blade positions in a shoot, sampling seasons and prolonged storage at subfreezing temperatures on the supercooling capability and survival are determined. The involvement of tissue intactness and tissue structure in the supercooling capability is also addressed. Finally, supercooling and freezing injuries of leaf blades in the field are also observed. Knowledge of these characteristics allow this plant to be used for studying the mechanisms of deep supercooling and for better understanding the role of this unique cold hardiness strategy in winter survival in the field.

## Materials and methods

### Plant materials

Culms with leaves were collected from populations of *Sasa senanensis* on the campus of Hokkaido University, Sapporo from August to December in 1982, 1983, 2006, and 2012 and immediately used for experiments. Experiments were conducted mainly with current year shoots and their leaf blades unless otherwise noted. New shoots usually emerged from early June until early July with all the leaf blades completing their unrolling and expansion by late July in the collection site.

### Determination of cold hardiness and freeze injuries by laboratory freeze tests

Five or more entire current year shoots or detached leaf blades (usually the second or third leaf blade from the top) were collected in different months and enclosed in polyethylene bags for each designated temperature. They were ice-inoculated with snow and kept at −5°C overnight, then cooled at 2°C/h (unless otherwise specified) to the designated temperatures (−5 to −30°C) and kept there for 4 h before being thawed at 2°C overnight. In some experiments, the shoots or leaf blades were stored at the designated temperatures for 1–14 days before being rewarmed at 2°C overnight. The shoots or leaves were incubated in polyethylene bags at room temperature for 1 week before the visual determination of injury. The injury of the leaf blades was rated between 0 (no injury) and 100% (totally killed) depending on the % area of the tissues showing necrosis (an instance is shown in **Figure 3F**). The injury rates of the leaf blades exposed to each designated temperature were averaged and plotted on a graph where LT_20_ or LT_50_, the lethal temperature at which 20% or 50% of the leaf blades got injured, were estimated.

### Differential thermal analysis

Leaf segments (3–4 cm long) excised from the second or third leaf blade on current year shoots of *S. senanensis* were moistened with a small amount of water on the adaxial surface and folded before being attached to the thermocouples by wrapping with aluminum foil. Then they were kept at −5°C overnight and cooled (without thawing) from −5°C down to −40°C at 2°C/h in a programmable deep freezer unless otherwise specified. Exothermic events were detected with a copper-constantan thermocouple attached to the leaf surface and amplified by a factor of 40–100 times prior to recording (Ishikawa and Sakai, 1982). Six to ten leaf segments were used for each determination. Similar cooling conditions were employed in DTA and laboratory freeze tests (detailed above) to allow direct comparison of the exotherms in DTA and the injury/survival data of laboratory freeze tests (Table 1). In other DTA experiments, samples with or without being moistened with water were cooled from 5°C to −40°C at 2°C/h. In experiments to determine the effect of freeze/thaw cycles on the supercooling capability, the leaf segment once used for DTA (frozen to −40°C) were thawed at 4°C and immediately used for another DTA run.

**Table 1:** **Seasonal changes in cold hardiness (LT_20_), initiation temperatures of the low temperature exotherm (LTE_i_) and water contents of *Sasa senanensis* leaf blades (2nd and 3rd leaves from the top on current year shoots)**.

**Date**	**Cold hardiness (°C)**	**LTE_i_ (°C)**	**Water content**
	**LT^a^_20_**		**(fr.wt. %)**
August 4	−11.0 ± 1.2	−12.8 ± 1.4	56.5 ± 1.8
September 26	−15.0 ± 0.9	−14.7 ± 1.0	55.5 ± 1.4
October 21	−15.6 ± 1.0	−16.4 ± 1.9	51.7 ± 1.2
November 8	−17.0 ± 1.4	−18.2 ± 2.2	49.4 ± 1.0
December 23	−20.2 ± 1.0	−20.4 ± 0.9	49.8 ± 1.3

*Specimens were kept at −5°C overnight (ice-inoculated) and cooled (without thawing) from −5°C to −40°C at 2°C/h both in DTA and freeze tests to make direct comparison allowable (for more details, refer to the Material and Methods section). The data are the mean ± SD (n = 6 ~ 10)*.

a *LT*_*20*_, *the lethal temperature at which 20% of the leaf blades were injured. This was estimated by plotting % injured leaf areas showing necrosis at each designated temperature*.

### Visual observation of *Sasa* leaf blades at subfreezing temperatures

As described in the previous paper (Ishikawa, 1984), the freezing of supercooled tissue units compartmentalized by the transverse and longitudinal veins can be readily recognized by the naked eye as blue spots during the freeze tests. Thus, leaf blades on the current year shoots cooled at 2°C/h were carefully observed or photographed in cold rooms set between −5 to −20°C and the percentage area of the freeze-spots that appeared was recorded. In some experiments, the leaves were kept at the designated temperatures for 4 h~7 days before being visually observed for the occurrence of freeze-spots.

### Cryomicroscopic observation of freezing process of *Sasa* leaf blades

The freezing process of leaf blade segments (5 mm square segments excised from the 2nd leaf blade from the top on a current year shoot collected in December) was observed under a cryomicroscope equipped with a cooling stage (LK-600PM, Linkam Scientific Instruments, UK) and recorded with an analog video camera. Photos or videos were captured with a computer and analyzed.

### Observation of deep supercooling and freezing injuries in *Sasa* leaves in the field

To verify whether wintering *Sasa* leaves under field conditions also remain supercooled, the shoots were collected on a −10°C night and brought to a cold room at −10°C without thawing. Then the leaves were carefully observed for the occurrence of freeze-spots (frozen units) and directly used for DTA without thawing. The leaves that were accidentally uncovered of snow were also observed on very cold mornings (−20°C or lower) to determine the occurrence of frozen spots on the leaves.

To detect freezing injuries in *Sasa* leaves in the field, the shoots of this species were carefully observed in March and April during the melt of the snow cover.

### Determination of water content

The dry weight of the leaf blades was determined gravimetrically after drying at 80 °C for 48 h. The water content was expressed as the percentage of fresh weight.

## Results and discussion

### Deep supercooling of *Sasa* leaf blades in regard to tissue units and their freezing behavior at non-lethal and lethal temperatures

The longevity of leaf blades of *S. senanensis* is usually 2 years and the leaf blades that newly appear in June ~ July are important for photosynthesis during the rest of the year and the following year except for the period of snow cover (Lei and Koike, 1998). In plants growing in the forest understory, the timing of greatest carbon gain is during early spring and late fall (leafless period of deciduous forests). Consequently, successful overwintering of the leaf blades is considered crucial for the ecological strategy of this plant.

**Figure 1:**
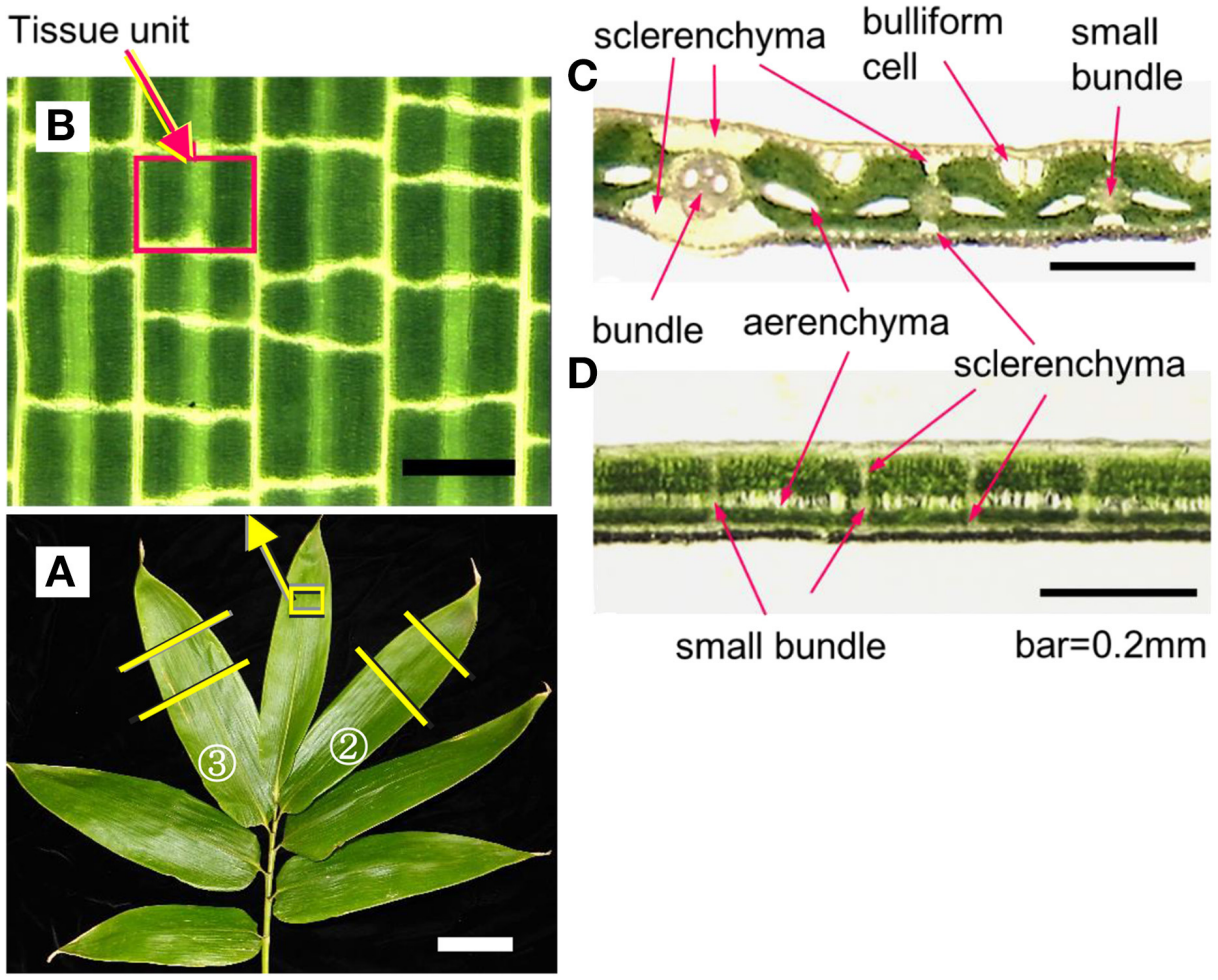
**An example of current year shoots of *Sasa senanensis* showing the adaxial surface (A) and microscopic view of the leaf blade, delineating a tissue unit compartmentalized with the longitudinal and transverse veins **(B)****. Microphotographs of transverse **(C)** and longitudinal **(D)** sections of current year leaf blades of *Sasa senanensis* (adaxial surface on the top). Scale bars in B, C, D = 0.2 mm, in A = 5 cm.

Leaf blades of *S. senanensis* have a ladder-like vein network and small pieces of tissues are compartmentalized with longitudinal and transverse veins (Figures 1A,B). Hereafter, we refer to these tissues as the tissue units or compartmentalized tissue units as they function as the units that remain unfrozen by supercooling, which will be detailed later. Both the longitudinal and transverse veins have thick-cell walled sclerenchyma cells developed on the adaxial and abaxial sides of the vascular bundles (Figures 1C,D), which separate the tissue units from the adjacent units in all directions. The extent of sclerenchyma development differs considerably depending upon the size and type of veins: major longitudinal veins (ribs) have more pronounced development whilst transverse veinlets usually have less sclerenchyma development. Mesophyll cells in a tissue unit are sandwiched by the epidermis and subtending hypodermis on both adaxial and abaxial sides whilst they contain the aerenchyma in the center.

**Figure 2:**
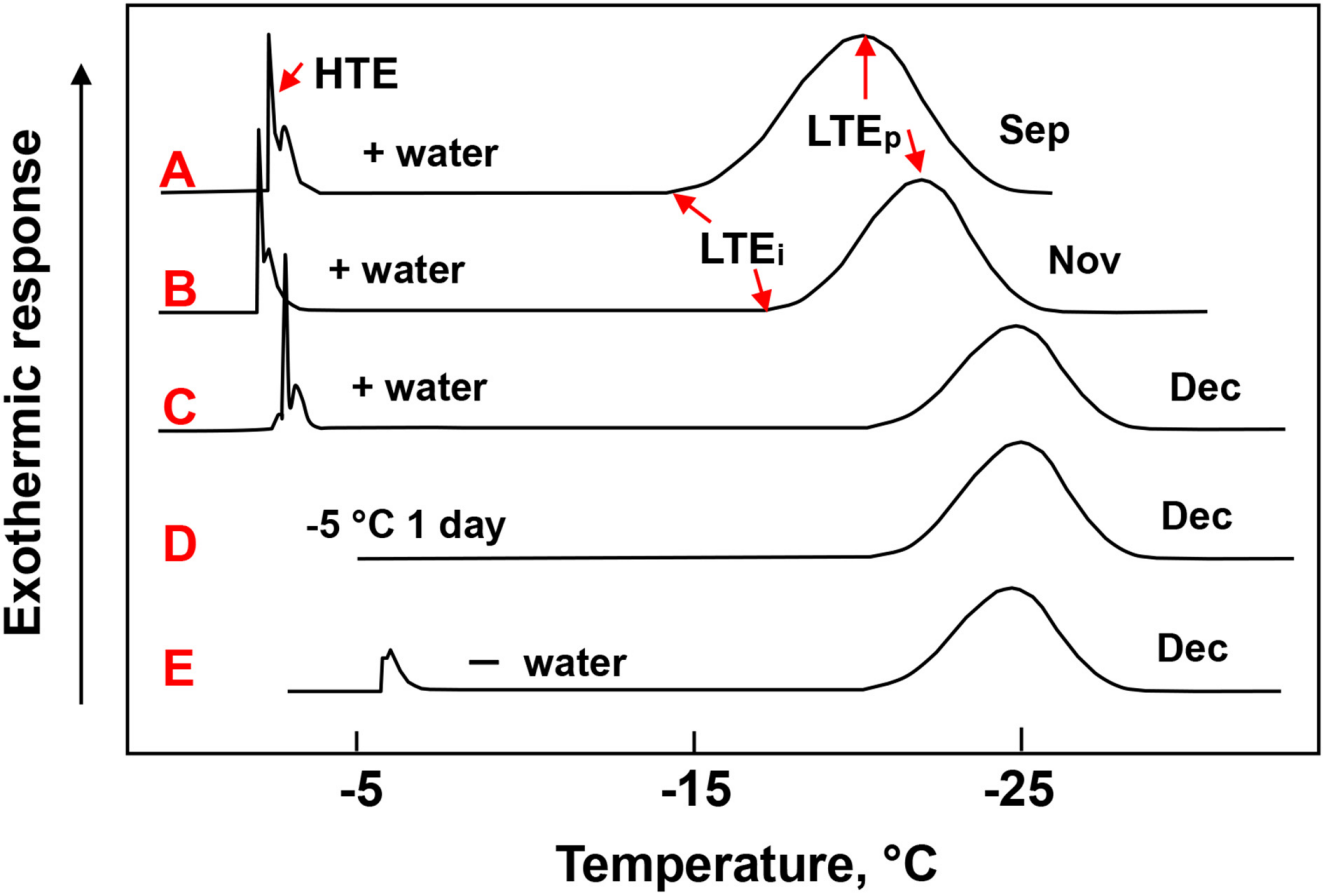
**Typical DTA profiles of leaf blade segments of *Sasa senanensis* current year shoots collected in different months**. Leaf blade segments (3–4 cm long) excised from the second or third blade from the top as shown in Figure 1A were used for DTA. The leaf segments, either following moistening with a small amount of water **(A–C)** or directly (without moistening) **(E)**, were cooled at 2°C /h from 5 to −40°C as detailed in the Materials and Methods section. In the case of **(D)**, the leaf segments moistened with water were stored overnight at −5°C before being further cooled for DTA without thawing the sample. HTE, high temperature exotherm; LTE_i_, the initiation temperature of low temperature exotherm (LTE); LTE_p_, the peak temperature of LTE.

When leaf blade segments collected in December were cooled in DTA, they showed a high temperature exotherm (HTE) at −5 ~ −6°C (Figure 2), which was shifted to higher temperatures (−2 ~ −4°C) when they were moistened with a small amount of water on the adaxial surface (Figure 2C) and disappeared when they were stored at −5°C overnight (Figure 2D). The HTE most likely represent the freezing of the water in the xylem vessels and the epidermis. This has been estimated from visually observing the slight changes in the surface leaf color and infra-red thermography studies (data not shown). It remains yet to be further confirmed with other methods such as cryo-SEM (scanning electron microscopy) and high resolution MRI (magnetic resonance imaging) (Ishikawa et al., 1997; Price et al., 1997).

**Figure 3:**
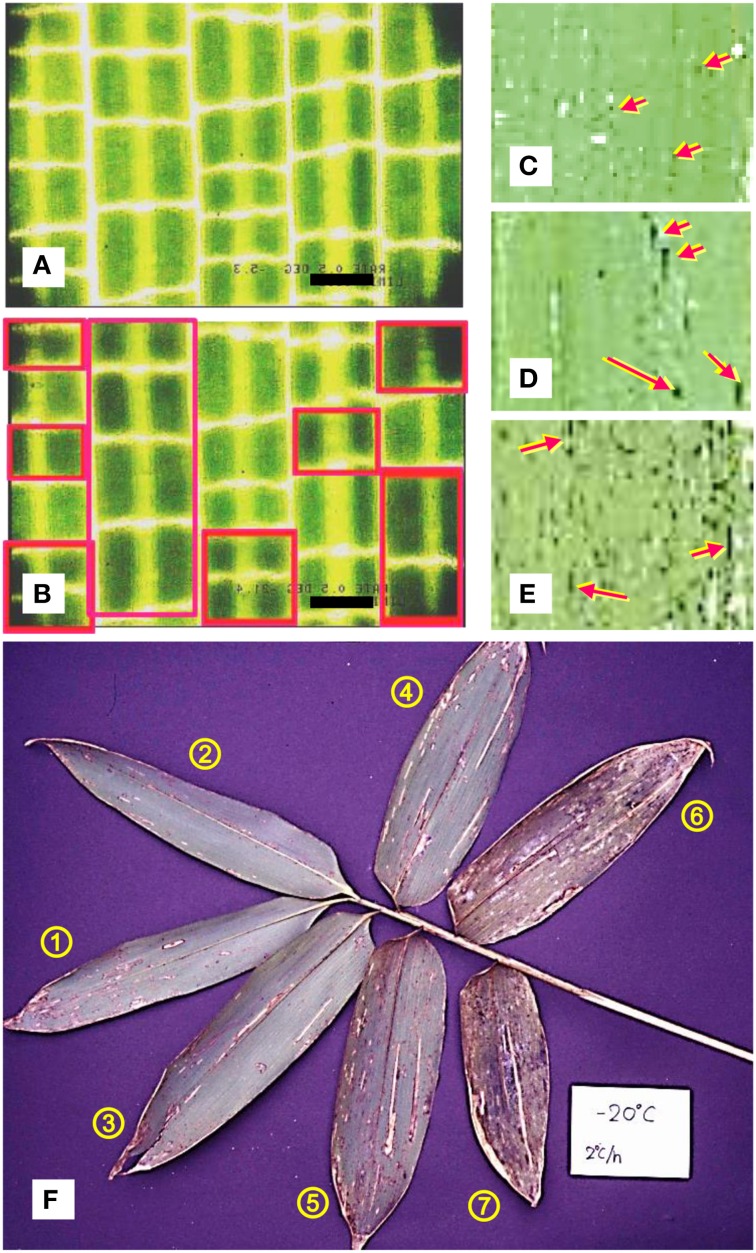
**Cryomicroscopic photos showing the freezing process of tissue units in current year leaf blades (5 mm square tissue segments) of *Sasa senanensis* collected in December (cooling rate: 0.5°C/min)**. Between −5 and −19°C, no freezing of tissue units were detected **(A)** whilst during cooling to −21.5°C, freezing of tissue units were clearly observed by the random darkening (each darkening comprising twice consecutive flashing) of the units **(B)**. Scale bar = 0.2 mm. Freeze-spots observed on the abaxial surface of the leaf blades stored at −20°C for 4 h **(C)**, 1 day **(D)** and 7 days **(E)** (the photos were taken in a cold room kept at −20°C without thawing the leaf blades). Typical injuries seen in *Sasa* leaf blades (showing the abaxial surface after a week of incubation at room temperature) on current year shoots cooled to at 2°C/h to −20°C and kept for 4 days **(F)**. The circled numbers besides the leaf blades indicate the leaf blade order (position) from the top leaf.

When the leaf blade segments (collected in December) were further cooled in DTA, they showed a LTE starting around −20°C and having a peak around −24°C (LTE_p_) (Figures 2C–E). The initiation temperature of LTE (LTE_i_) was in agreement with the LT_20_ (lethal temperature at which 20% of tissues were injured). The LTE_i_ is often unclear due to a very gradual exothermic rise making it difficult to accurately determine from the DTA profiles (Figure 2). This often results in slight differences in the LTE_i_ and LT_20_. The LTE in the DTA profiles corresponded closely to the sudden darkening of the tissue units detected under cryomicroscopy, which likely indicates the freezing of the supercooled units (Figure 3B). Closer observation revealed that the darkening (freezing) of a unit always occurred twice in a consecutive manner: the second occurrence following the first after a few seconds or less and each darkening completing in less than a second (flash type of freezing). This presumably arises from the structure of the unit: the mesophyll cells in a unit are divided into the upper and lower groups, separated by the aerenchyma tissues in between (Figures 1C,D). The successive darkening (flashing twice) of each unit most likely represents the freezing of the upper and lower mesophyll cell groups. As the temperature further decreased, more tissue units froze randomly and were also recognized as blue freeze-spots (more easily visible from the abaxial side) by the naked eye when a group of neighboring tissue units were nucleated (Figures 3D,E).

The results indicate that the tissue units in leaf blades remain unfrozen until −20°C by supercooling in spite of the neighboring xylem vessels and the epidermis being likely frozen. The sudden freezing (flashing twice) of supercooled units likely caused intracellular freezing (yet to be verified) of the live cells (mostly mesophyll cells) in the units, which resulted in the lethal injury of the mesophyll cells in the tissue units. Each flash freezing likely represents the simultaneous intracellular freezing of the upper or lower layer of mesophyll cells and the aerenchyma tissues are unable to function as effective ice barriers. In *Sasa* leaf blades, the breakdown of deep supercooling occurs at the tissue level with the small compartmentalized tissue working as the unit of freezing. The cells in a tissue unit share the common destiny (alive or dead) until the last moment of flash freezing (breakdown) of the supercooled unit. This is in contrast to the known mechanism of deep supercooling in the xylem ray parenchyma cells: individual cells work as the unit of deep supercooling and the breakdown of supercooling occurs at the individual cell level (Hong and Sucoff, 1982; Neuner et al., 2010). In deep supercooling or extraorgan freezing of flower buds, the lethal breakdown of supercooling occurs at the organ level (i.e., cells in the entire flower primordium or floret freeze almost simultaneously) and the floret functions as the unit of supercooling/breakdown (Ishikawa and Sakai, 1981; Ishikawa et al., in press).

The leaves of *Cinnamomum* (cold-tender tropical plant) display scattered or discontinuous type of freezing between −7 and −10°C, which often allows the leaf portions to survive by temporarily avoiding lethal freezing (Sakai and Larcher, 1987; Hacker and Neuner, 2007). The lethal freezing (likely intracellular) proceeds in small leaf tissue patches (a group of leaf cells freeze simultaneously), which is seemingly similar to the freezing behavior of *Sasa* leaf blades. Yet in the *Sasa* leaf blades, the epidermis and xylem vessels freeze autonomously in an uninterrupted manner by −6°C (Figure 2), which is not lethal while the mesophyll cells remain supercooled until −15 ~ −20°C, whose lethal breakdown proceeds with the compartmentalized tissue as the unit. Each freezing event in both species (precise localization and whether it is intracellular or intercellular freezing) remains to be verified with cryo-SEM and high resolution MRI.

### Stability of supercooling in the tissue units and the importance of ice barriers

The freezing of the tissue units was primarily governed by the temperature as shown by the constant rate cooling experiments (Figures 2, 3B). It also depended on the exposure time (Figures 3C–E) when the leaf blades were exposed to the threshold temperature range (−15 ~ −20°C) whilst it was marginally affected by prolonged exposure at −5 ~ −10°C (Table 2, Figure 2D). This is similar to the freezing of fine water droplets which show both temperature-dependent and time-dependent behavior, the latter of which is evident at −18°C but not at −12°C (Vali and Stansbury, 1966; Vali, 1971).

**Table 2:** **Effects of prolonged storage at −5°C with ice on the survival of current year leaf blades collected in summer and winter**.

**Days at −5°C**	**Injury rating (0–100%)**	
	**Summer leaf blades (August 9)**	**Winter leaf blades (January 8)**
1	6.3 ± 1.2	0
4	45.0 ± 2.8	0
9	82.5 ± 4.1	ND
14	ND	0
0°C for 12 days	0	0

*Current year shoots were ice-inoculated with snow and stored at −5°C in polyethylene bags for 1–14 days. No or only marginal freezing of supercooled tissue units were observed during 14 d of storage at −5°C in summer and winter leaf blades. The data are the mean ± SD (n = 4)*.

Regardless of temperature- or time-dependent breakdown of supercooling, the freezing of tissue units proceeded in a random manner, yet more likely in the longitudinal direction rather than in the transverse direction (Figures 3B–D). This may be related to the smaller sclerenchyma development associated with the transverse veins compared to the longitudinal veins (Figures 1C,D), given that they work as the ice barrier which prevents freezing of a tissue unit from propagating into the neighboring tissue unit.

Interestingly, leaf blades of tropical woody bamboo species such as *Bambusa*, which are susceptible to freezing by −5°C, do not have a distinct LTE although the HTE is distinct. Moreover, they totally lack the development of sclerenchyma associated with transverse veins (the vein network is not like ladder as shown in Figures 1, 3, but mainly comprises parallel longitudinal veins). In *Bambusa* leaf blades, once the freezing of mesophyll tissues in between the veins was initiated, it proceeded freely along the longitudinal veins, which likely resulted in injury (Ishikawa, unpublished). This supports the hypothesis that the development of sclerenchyma is important for maintaining the supercooling of the tissue unit by preventing the freezing from spreading into the neighboring unfrozen tissue units.

Together the results demonstrate that the small tissue unit compartmentalized by the longitudinal and transverse veins functions as the unit of remaining unfrozen until −15 ~ −20°C by stable deep supercooling. Since the xylem vessels in the vascular bundles and epidermis likely freeze at high subzero temperatures (−2 ~ −6°C) as represented by HTE, the cell walls of the xylem vessels or adjacent tissues in the vascular bundles (such as bundle sheath) and the cell walls of the epidermis or the hypodermis (adjacent to epidermis) may function as the barrier against ice intrusion from the already frozen tissues into the unfrozen units. The group of sclerenchyma cells with very thick cell walls developed beneath and above the longitudinal and transverse veins (Figures 1C,D) allow the tissue units separated from each other to prevent the freezing of a unit from propagating into the adjacent unfrozen units. Theoretically, the mesophyll cells enclosed in a tissue unit chamber need barriers (structural or biochemical) against ice intrusion on six sides (chamber walls). Simultaneously, they must develop some biochemical mechanism(s) to increase the capability and stability of supercooling to remain unfrozen within the tissue unit (Ishikawa et al., 2009).

The results also demonstrate that the compartmentalized tissue surrounded by the longitudinal and transverse veins works as the functional unit at the tissue level. The cells in the tissue unit share a common destiny (survival or injury) in response to subfreezing temperatures (deep supercooling or freezing). The compartmentalized tissue may also function as the unit of physiological responses to other abiotic and biotic stresses such as hypersensitive responses against pathogens or insects. This remains to be confirmed by the experts of respective fields.

### Leaf order (position) dependent variation in cold hardiness levels

**Figure 4:**
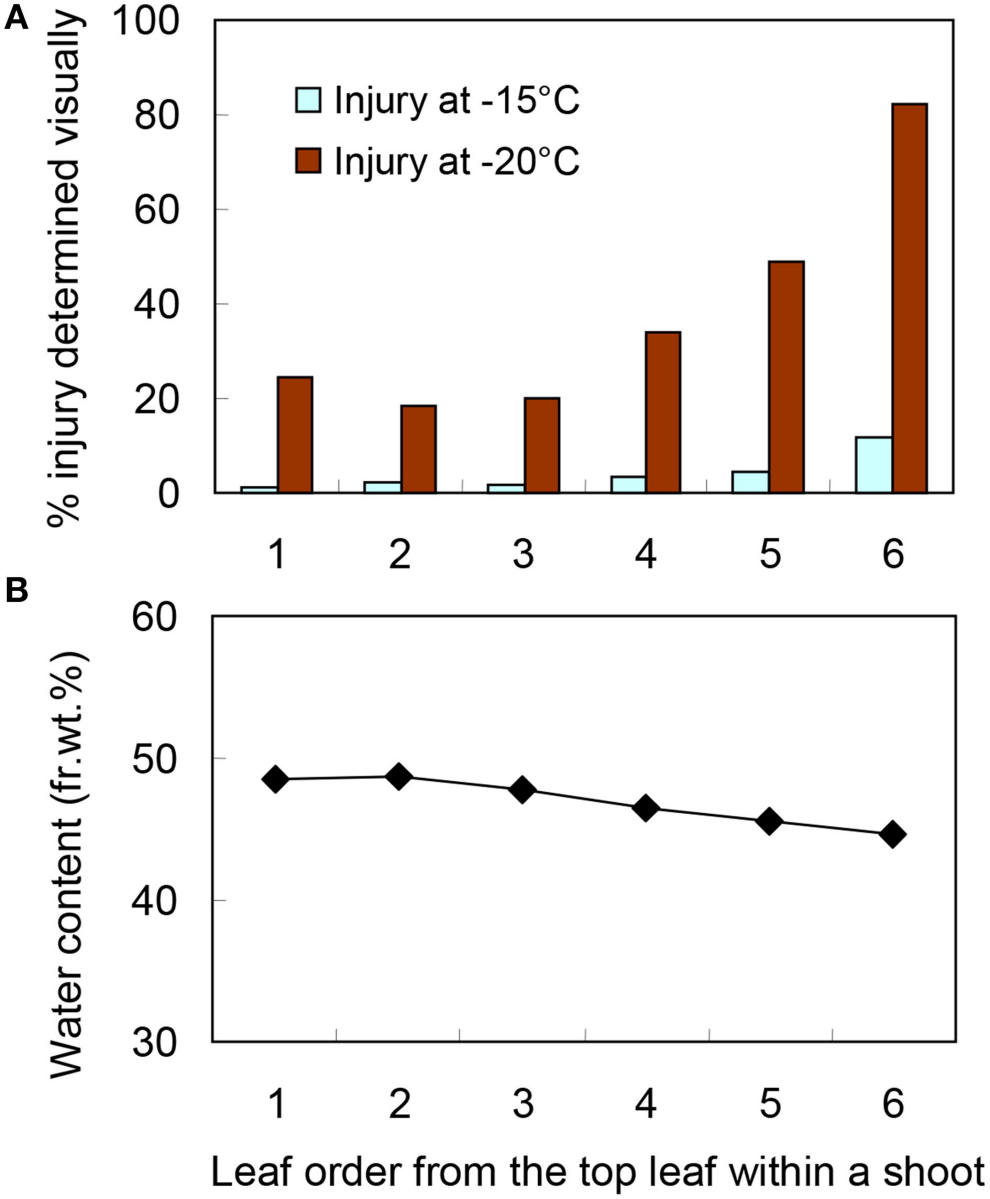
**The extent of freezing injury (A) and water content (B) varied depending on the leaf blade position within wintering current year shoots of *S. senanensis* collected in December**. Freezing injury was determined by visually rating (0 ~ 100%) the injured leaf area showing necrosis. The numbers on the abscissa are the leaf blade order (position) from the top leaf. The current year shoots were kept at −5°C overnight with snow, then cooled at 2°C/h to −15 and −20°C and left there for 4 days before being thawed at 4°C (a typical example shown in Figure 3F).

There were obvious differences in the level of leaf blade freezing injury within a current year shoot collected in December (Figure 3F) when they were slowly cooled and exposed to −20°C for 4 days. Leaf blades positioned second or third from the top suffered the least injury while there were increasing levels of injury from the fourth to sixth leaf blades (Figure 4A). A similar tendency was observed but differences in injury levels were much smaller when they were exposed to −15°C for 4 days. Since only marginal blue freeze-spots were detected in leaf blades exposed to −15 or −20°C for 1 ~ 4 h, the results indicate that the supercooling was more stable at −15°C than at −20°C and less stable in the leaf blades from lower positions in a shoot. The water content was lower in the leaf blades of lower positions (Figure 4B), which did not result in increased supercooling capability (Figure 4A). This is contrary to the seasonal decreases in the water content in leaf blades from August to December linked to the increased supercooling capability (Table 1). This is also different from the supercooling capability of azalea florets (Ishikawa and Sakai, 1981; Ishikawa et al., in press) and hydrated seeds (Junttila and Stushnoff, 1977; Ishikawa and Sakai, 1978), where the reduced water content results in enhanced supercooling capability. The varied levels of supercooling stability associated with leaf order (position) could either be due to differences in the cellular content and/or the quality of ice barriers against intrinsic nucleation (from the adjacent frozen units) or extrinsic nucleation, which remain to be elucidated.

The results clearly show that the second and third leaf blades suffer the least injury with only marginal injury variance observed within an individual leaf blade (Figure 3F). Thus, these are considered suitable for cold hardiness mechanism studies and used in experiments on supercooling capability (Tables 1, 3, Figure 2).

**Table 3:** **Effect of freeze-thaw cycles on the supercooling capability in *Sasa* leaf blades of different months**.

**Collection date**	**Intact (1st run)**		**−40°C/thaw (2nd run)**		**Water content**
	**LTE_i_ (°C)**	**LTE_p_ (°C)**	**LTE_i_ (°C)**	**LTE_p_ (°C)**	**(fr.wt. %)**
September 20	−14.1 ± 0.1	−19.6 ± 0.4	−11.4 ± 0.3	−15.9 ± 0.3	56.8
			−9.7 ± 1.1^a^	−15.4 ± 0.4^a^	
November 4	−17.6 ± 0.3	−21.2 ± 0.5	−13.2 ± 0.8	−16.8 ± 0.8	52.8
December 20	−20.3 ± 0.4	−24.8 ± 0.2	−14.8 ± 0.6	−20.2 ± 0.8	49.8

*The leaf segments (3–4 cm) were moistened with a small amount of water and cooled at 2°C /h from 5 to −40°C for DTA. The supercooling capability was expressed as the mean ± SD of the initiation (LTE_i_) and peak (LTE_p_) temperatures of low temperature exotherms detected (n = 4-6). The samples used in the first DTA run were thawed at 4°C and immediately used for the second DTA*.

a *Similarly the third DTA run was conducted on September samples*.

There is a seeming discrepancy in the levels of cold hardiness in leaf blades (LT_0_, the lowest temperature that can be tolerated without injury: −20°C vs. −15 ~ −17°C) between our previous determination (Ishikawa, 1984) and the one by Konno and Sakai (1975). This could be partly attributed to the differences in the levels of cold hardiness depending upon the leaf blade position. It may also arise from the instability of deep supercooling at the threshold temperatures (−15 ~ −20°C), since in our determination, the leaves were exposed to the designated temperatures for 4 h whilst in the determination by Konno and Sakai (1975), they were exposed for at least 24 h.

### Seasonal changes in cold hardiness, supercooling capability and water content of *Sasa* leaf blades

To check whether the leaf blades undergo seasonal changes in the level of cold hardiness, laboratory freeze tests and DTA were conducted with the 2nd and 3rd leaf blades on current year shoots collected from August until December. The results show that current year leaf blades increased cold hardiness levels from August (LT_20_ = −11°C) to December (LT_20_ = −20°C) and underwent cold acclimation (Table 1). During this period, there were concomitant increases in the supercooling capability (Figures 2A–C). The LT_20_ values approximately coincided with the lowering LTE_i_ detected in DTA (Table 1). During this period, leaf blade water content decreased from 56.5% fr. wt. (August) to 49.8% (December). This is obviously different from the case of *Trachycarpus fortunei*, which is another typical supercooling plant, but shows only marginal seasonal changes in the supercooling capability and maintains the same level of cold hardiness (LT_0_: −13 ~ −14°C) in the leaf blades throughout the year (Larcher and Winter, 1981; Larcher et al., 1991). The observed seasonal changes in the deep supercooling capability in *Sasa* leaf blades are similar to the seasonal changes in the deep supercooling capability of xylem ray parenchyma cells of temperate woody species such as apple (Quamme et al., 1972).

### Effect of prolonged storage at −5°C on the supercooling and survival of summer and winter leaf blades

To check the effect of prolonged storage of *Sasa* leaves at high subzero temperatures, the current year shoots collected in August and January were placed in polyethylene bags with a small amount of snow to ensure ice-inoculation and to avoid desiccation. Subsequently, they were stored at −5°C for 1–14 days. Even after 14 days of storage at −5°C, both summer and winter leaf blades looked normal (no desiccation injury) though the epidermis and xylem vessels were likely kept frozen as indicated by their slightly darker color. Careful observation at −5°C revealed that no or only marginal freezing (blue spots observable on the abaxial side of the leaf blades) of compartmentalized tissue units were detected during 14 days of storage at −5°C irrespective of the collection season. However, when they were thawed and incubated at room temperature for 1 week, only summer leaves exhibited injuries that increased with the longer incubation at −5°C. In contrast, the leaves stored at 0°C with snow for 12 days suffered no such injuries regardless of the collection season (no visible chilling injury occurred after prolonged exposure to 0°C). This implies that the injury observed in the summer leaves is a novel type of low temperature-induced injury that occurs in tissues which remain unfrozen (supercooled) at −5°C for a prolonged period. This is likely due to the imbalance caused by the condition where the epidermis and xylem vessels freeze while the remaining live cells stay unfrozen rather than a simple metabolic imbalance caused by exposure to a lower temperature (−5°C).

### Effect of freeze-thaw cycles on the supercooling ability of leaf blades

To check the contribution of tissue intactness and viability on the supercooling capability, the leaf blade segments used for DTA (after a freeze-thaw cycle) were immediately used for another DTA run (Table 3). Both LTE_i_ and LTE_p_ were shifted to higher temperatures by 3–5°C, irrespective of the month of collection. When this treatment was repeated twice in September leaf blades, the LTE_i_ was further shifted to higher temperatures whilst there was no further change in the LTE_p_. Similar results were obtained in the supercooling capability in leaf blades of *Trachycarpus fortunei* (Ishikawa et al., 2009). Since a freeze-thaw treatment (−40°C/4°C) is lethal and the results imply the importance of tissue intactness in the supercooling capability of the leaf blades. The contribution of the tissue intactness may involve several factors, such as cellular protoplasm content, intactness of ice barriers and fine distribution of water within the tissues, which remains to be further investigated.

### Observation of freezing injuries of *Sasa* leaves in the field

When wintering current year shoots of *Sasa* (uncovered with snow) were collected on −10°C nights and brought to a cold room (set at −10°C) without thawing, the occurrence of blue freeze-spots (frozen units) on either side of the leaf blades could not be detected. When the leaf blade segments were carefully excised and directly used for DTA without thawing, DTA profiles similar to Figure 2D were obtained. When *Sasa* leaves accidentally uncovered with snow were observed on very cold nights (−20°C or lower) in mid-winter under flashlight, numerous blue freeze-spots (frozen tissue units) on the leaves, similar to the ones shown in Figure 3E were observed. These observations clearly demonstrate that the deep supercooling capability in *Sasa* leaf blades function as the cold hardiness mechanism in the field as well and the breakdown of supercooling resulted in the injuries *in situ*.

When the *Sasa* leaf blades accidentally uncovered with snow and exposed to −20°C or lower were observed in the spring time, they turned whitish yellow (seemingly desiccated) and the freeze-spots or injuries as shown in Figure 3F were not readily distinguishable. It is possible that frozen tissue units in the leaf blades may no longer be able to retain water within the tissues.

Endo and Akitaya (unpublished) observed similar severe leaf freezing injuries of *Sasa* plants occurring on steep slopes which got accidentally uncovered by snow during snow avalanches and exposed to extremely low temperatures (−20°C or lower) in central Hokkaido.

As mentioned earlier, current year leaves of *S. senanensis* have a longevity of 2 years and are important for carbon gain in late fall and early spring of the following year. Therefore, wintering performance is considered crucial in establishing their dominance in the forest understory. The deep supercooling capability of leaf blades most likely serves as the cold hardiness mechanism to survive subfreezing temperatures *in situ* in late fall, early winter and spring time when the plants are not covered with snow. Yet the stability of supercooling in leaf blades is likely insufficient to withstand the low temperature extremes (−20°C or lower) and to successfully overwinter in Hokkaido (see the supplementary file). This may have allowed the plants to become recumbent under snow cover to avoid serious losses due to freezing injury. This is probably the reason why this species is located in an area covered by more than 50 cm of snow in midwinter. Similar reasons for the snow-recumbent strategy are expected in *Sasa kurilensis*, which occurs at higher elevations and has 2–5°C higher levels of cold hardiness than *S. senanensis* in laboratory freeze tests (Konno and Sakai, 1975; Ishikawa unpublished). In contrast, *Sasamorpha borealis*, which occurs in the area with snow deposits of less than 50 cm (Suzuki, 1961), seems to have developed a different strategy to survive the cold winter. The leaf blades of this species tolerated −25°C without any injury in laboratory freeze tests and this is likely the most cold hardy bamboo species (Konno and Sakai, 1975; Ishikawa unpublished). This species has stiff and upright culms with reduced elasticity compared to *S. senanensis* and usually does not become recumbent under snow in their natural habitat. Their leaf blades are smaller and thicker with increased longevity (2–3 years) and the tissue units are compact and rigid, which is likely linked to the increased stability of supercooling (Ishikawa unpublished).

In conclusion, leaf blades of *S. senanensis* owe their cold hardiness mechanism to the capability to deeply supercool not only in laboratory conditions but also in the field. The small tissues compartmentalized with longitudinal and transverse veins served as the units that remained unfrozen by supercooling and flash freezing of these supercooled units was lethal. The supercooling of these tissue units was stable at −5 ~ −10°C but prone to be less stable during prolonged exposure to −20°C and especially in the leaf blades of lower positions. The leaf blades showed seasonal increases in the supercooling capability and corresponding increases in the cold hardiness levels (cold acclimation) from summer to winter. The leaf blades are a good material to study both the biochemical and structural mechanisms involved in deep supercooling, such as protoplasmic contents and ice barriers. The leaf blades are translucent and their freezing process are readily visible under cryomicroscopy. Since the majority of living cells remain deeply supercooled, it may be easier to assign the substances involved in the deep supercooling capability and its stability from the extract of the tissues. We also found a novel type of low temperature-induced injury in the tissue units that remained unfrozen (supercooled) at −5°C for a prolonged period, which only occurred in summer leaves. It was postulated that development of sclerenchyma on both sides of longitudinal and transverse vascular bundles is an important factor contributing to the establishment of deep supercooling in the tissue units.

### Conflict of interest statement

The authors declare that the research was conducted in the absence of any commercial or financial relationships that could be construed as a potential conflict of interest.

## Abbreviations

DTA: differential thermal analysis
HTE: high temperature exotherm
LT_20_: the lethal temperature at which 20% of the leaf blades get injured
LTE: low temperature exotherm
MRI: magnetic resonance imaging
SEM: scanning electron microscopy.
